# High-resolution SNP array analysis of patients with developmental disorder and normal array CGH results

**DOI:** 10.1186/1471-2350-13-84

**Published:** 2012-09-17

**Authors:** Linda Siggberg, Ala-Mello Sirpa, Linnankivi Tarja, Avela Kristiina, Scheinin Ilari, Kristiansson Kati, Lahermo Päivi, Hietala Marja, Metsähonkala Liisa, Kuusinen Esa, Laaksonen Maarit, Saarela Janna, Knuutila Sakari

**Affiliations:** 1 Department of Pathology, Haartman Institute, University of Helsinki, and Laboratory of Helsinki and Uusimaa University Hospital, Helsinki, Finland; 2 Rinnekoti Foundation, Rehabilitation Home for Children, Espoo, Finland; 3 Department of Pediatric Neurology, Helsinki University Central Hospital, Helsinki, Finland; 4 Väestöliitto, The Family Federation of Finland, Department of Medical Genetics, Helsinki, Finland; 5 Department of Pathology, VU University Medical Center, Amsterdam, The Netherlands; 6 Public Health Genomics Unit, Department of Chronic Disease Prevention, National Institute for Health and Welfare, Helsinki, Finland; 7 Institute for Molecular Medicine Finland FIMM, University Helsinki, Helsinki, Finland; 8 Department of Clinical Genetics, Turku University Hospital and Department of Medical Biochemistry and Genetics, University of Turku, Turku, Finland; 9 Department of Pediatrics, Satakunta Hospital District, Pori, Finland; 10 Population Health Unit, Department of Health, Functional Capacity and Welfare, National Institute for Health and Welfare, P.O. Box 21 00014, Helsinki, Finland

**Keywords:** Developmental disorder, SNP array, Diagnostic yield

## Abstract

**Background:**

Diagnostic analysis of patients with developmental disorders has improved over recent years largely due to the use of microarray technology. Array methods that facilitate copy number analysis have enabled the diagnosis of up to 20% more patients with previously normal karyotyping results. A substantial number of patients remain undiagnosed, however.

**Methods and Results:**

Using the Genome-Wide Human SNP array 6.0, we analyzed 35 patients with a developmental disorder of unknown cause and normal array comparative genomic hybridization (array CGH) results, in order to characterize previously undefined genomic aberrations. We detected no seemingly pathogenic copy number aberrations. Most of the vast amount of data produced by the array was polymorphic and non-informative. Filtering of this data, based on copy number variant (CNV) population frequencies as well as phenotypically relevant genes, enabled pinpointing regions of allelic homozygosity that included candidate genes correlating to the phenotypic features in four patients, but results could not be confirmed.

**Conclusions:**

In this study, the use of an ultra high-resolution SNP array did not contribute to further diagnose patients with developmental disorders of unknown cause. The statistical power of these results is limited by the small size of the patient cohort, and interpretation of these negative results can only be applied to the patients studied here. We present the results of our study and the recurrence of clustered allelic homozygosity present in this material, as detected by the SNP 6.0 array.

## Background

The diagnostic yield of microarray comparative genomic hybridizations (array CGH) has already proven to exceed that of cytogenetic methods, except when it comes to balanced rearrangements. A consensus statement suggests that microarrays should be used as the first line of testing for developmental disorders of unknown cause [1]. However, as our previous study shows, approximately 80 % of patients with a developmental disorder of unknown cause (mental retardation and/or malformations and/or neurological disorders) remain undiagnosed even by array CGH analysis (44 K, 180 K, or 244 K) [2]. Thus, other methods are clearly needed to define the pathogenic mechanisms. It is plausible that small copy number variants (CNVs) may go undetected if the probe coverage is limited, as it may be in low-resolution arrays. By increasing the resolution, one would thus expect to detect increasingly smaller pathogenic CNVs.

The frequency of uniparental disomy (UPD) in newborns is reportedly ~0.029% [3]. Around 1,100 cases of whole chromosome UPD and some 120 reports on segmental UPD are described in the literature [4]. Some recessive diseases are expressed in children who have inherited the mutation form a single carrier parent [5]. The explanation for this is a meiotic or very early mitotic recombination event between parental homologous chromosomes causing segmental UPD of the genomic segment containing the mutation, and thereby causing a reduction to homozygosity.

As the market is flooded with new arrays, most having an increased resolution and a promise of ever higher detection rates, the question remains what the added value is of these ultra-high resolution arrays. To test this, we used an array with 1.8 million probes and an average resolution of 0.7Kb to analyze samples of 35 patients with developmental disorder of unknown cause, normal karyotype, and normal array CGH results by use of Agilent 44 K, 180 K, or 244 K platforms (Agilent Technologies, Santa Clara, CA, USA).

## Methods

### Participants

Patients with previous normal array CGH results were asked to participate in the project. All 35 patients were Finnish of origin and had mild to severe mental retardation, associated with dysmorphic features and/or congenital anomalies (Table 1). In addition, 16 patients also had epilepsy. For diagnostic purposes patients had previously been analyzed by whole-genome array CGH (Human Genome CGH Microarray, Agilent Technologies, Santa Clara, CA); 20 using the 244 K platform, 8 using a 180 K platform, and 7 using a 44 K platform. Informed consent was given by all participating families. Blood samples were collected from all patients and their parents. Ethical permission for this project was given by the Ethics Review Board of Helsinki and Uusimaa Hospital District.

**Table 1:** Clinical characteristics of patients studied

**P. Nr.**	**Growth**	**Head and neck**	**Eyes and vision**	**Ears and hearing**	**Face**	**Cardiovascular**	**Genitourinary**	**Skeletal and limb defects**	**Neurologic**	**Other**
1.	Obesity		Astigmatism					Postaxial polydactyly (one foot) Short meta-carpals V finger clino-dactyly	ID Hypotonia	Bardet-Biedl suspected
2									Severe DD No walk/crawl No speech Epilepsy Drooling	
3.		Dolicocephaly Narrow, prominent forehead Low, uneven hairline	Epichantal folds						DD Abnormal pons	Hemangiomas
4.	Short stature				Low nasal bridge		Horseshoe kidney Anal atresia	Small hands and feet	ID	Balanced t(X;13)(q28;q12)
5.				Simple ears	Thick and straight eyebrows Broad nasal bridge Long philtrum Retrognathia				Autism ID Epilepsy	
6.	Short stature	Microcephaly	Blindness Optic nerve hypoplasia					Scoliosis	ID Epilepsy Severe hypotonia	
7.		Hydrocephalus							Brain malformation Severe DD	
8		Microcephaly	Hypertelorism		Small nose Low nasal bridge Tented upper lip				Severe DD Severe epilepsy	ATRX suspected
9.			Severe optic atrophy Impaired vision						ID Epilepsy Cortical atrophy	
10.			Mild hypertelorism	Low-set ears	Triangular face Small jaw High palate Thin upper lip			Hyper-extensible joints	ID Autistic features Intractable epilepsy	Frax-dna, SCN1A, CLN8 4p-FISH normal
11.			Upslanting palpebal fissures	Large earlobes	Small jaw			Clubfoot	ID Autistic features Intractable epilepsy	
12.			Epichantic folds	Large earlobes	Flat face			Tapering fingers	ID Autistic features ADHD	Balanced t(2;9)(q13q22.3) de novo
13.		Macrocephaly							Severe ID Hypotonia Autism Epilepsy	Inv 2(p13p25) mat., DMPK mutation negative
14.			Severe myopia Cataracta		Synophrys Curved eyebrows Upturned pinched nose Big mouth Full lips	Atrial septum defect			Severe DD Epilepsy	
15.		Microcephaly	Impaired vision			Ventricular septum defect			Epilepsy DD	
16.									ID Beahvioural disturbances Autism	No malformation or dysmorphism
17.									ID DD	
18.	Growth retardation		Hypertelorism			Mild ventricular septum defect			ID	
19. & 20.									DD	No structural defects
21		Microcephaly	Strabismus	Missing lobuli	Small nose Low nasal bridge Smooth philtrum Thin lips			Proximal thumbs Pes planus	ID Intractable epilepsy Ataxia	
22.	Pre- and postnatal growth retardation				Broad nasal root Short nose Bifid nasal tip		Cryptorchidism Hypoplastic scrotum	Scoliosis Syndactylies	Slow motor development Hypotonia Expressive language disorder	Congenital contractures Dimples
23.					Mild dysmorphism				ID Epilepsy	
24.		Microcephaly	Hypertelorism Epicanthic folds Disorder of visual cortex	Low-set and posteriorly rotated ears	Micrognathia Cleft palate				ID Epilepsy Hypoplastic cerebellar vermis	Monozygotic twin, twin sister healthy
25.	Tall stature Advanced bone age		Deep set eyes Hypotelorism Epicanthic folds Strabismus		Short nose Anteverted nares Tented upper lip		Cryptorchidism		ID No speech Autism	Glypican-3 and PHF6 mutation analyses negative
26.	Short stature								ID Intractable epilepsy Tremor Myoclonias Distal spasticity	
27.	Small for Gestational Age		Downslanting palpebral fissures Strabismus		Frontal bossing			Exostosis (familial) Broad hallux Overriding toes Scoliosis	ID Epilepsy	Inguinal hernia
28.			Downslanting palpebral fissures	Hearing impairment	Coarse hair Thick eyebrows Thick lips Malposition of teeth	Hypertrophic cardiomyopathy		Hip dis-placement Long thin bones	Normal intelligence	
29.									Epilepsy ID Alternating hemiplegia of childhood	
30.	Small for gestational age Prematurity Short stature	Microcephaly	Severe myopia Coloboma of papillae Optic atrophy Nystagmus Strabismus		High palate Gum hypertrophy	Coarctation of aorta	Inguinal hernia		ID Intractable epilepsy Hemiparesis (peri-ventricular leukomalacia)	
31.			Central blindness Nystagmus						ID Intractable epilepsy Hypotonia Distal spasticity	
32.	Short stature								Normal development	Vomiting Feeding difficulties
33.		Neck fistula			Dysmorphic malocclusion of teeth	Uni-ventricular heart			Brain atrophy Epilepsy	Simian-crease Sinus pilonidalis
34.									ID Intractable epilepsy Hypotonia Distal spasticity	
35.		Dolicocephaly	Epichantic fold	Simple ears	Thin upper-lip Long philtrum Broad nasal bridge				ID Arnold Chiari malformation	

The table presents the clinical characteristics of the 35 patients studied. DD = developmental delay, ID = intellectual disability.

### SNP array

DNA was extracted from blood samples according to standard protocols. Analysis by the Genome-wide human SNP array 6.0 was performed according to manufacturer protocols (Affymetrix, Santa Clara, CA, USA). In short; DNA was digested, ligated to adapters, and amplified by PCR. Samples were purified using magnetic beads and further fragmented and labelled with biotin. After hybridization arrays were washed and stained with streptavidin and anti-streptavidin antibodies and finally the arrays were scanned using the Affymetrix GeneChip scanner.

### Analysis

Data was extracted from the scanned image using the Genotyping console software V.3.0.2, creating a CEL file. Areas containing CNVs and allelic homozygosity were detected using the Hidden-Markow-Model. The resulting data was analyzed using the Chromosome Analysis Suite software V.1.0.

### Reference data

Data was extracted by comparison to a reference data set established from 90 Caucasian individuals, which had previously been analyzed using the SNP 6.0 array in the HapMap project (http://www.hapmap.org). As an additional in-house reference set, we used results of 54 individuals studied using the SNP 6.0 array, whereof 19 healthy normal relatives of the patients, and 35 unrelated patients with an unexplained developmental disorder. Sample identities were kept anonymous and the information was only used for reference purposes. These in-house reference sets were used to filter out polymorphic changes in the patient data studied here.

Selected CNVs of the patients were compared to a Finnish population cohort [6]. This population cohort data consist of CNVs detected, using whole-genome SNP analysis, in 2163 healthy Finnish individuals with PennCNV [7]. In the population data, low quality samples (N = 98) with Log R Ratio standard deviation of probe signal intensities > 0.35 or > 115 CNV calls were excluded. Only CNVs with three or more probes were included in the final population data. CNV calls of the study samples were clustered into CNV regions when individual CNVs overlapped by one or more base pairs.

### Filtering relevant CNVs and potential UPDs

The first set of default filtering marked all duplications and deletions ≥0.7Kb and all allelic homozygosities ≥100Kb containing at least 10 markers to be included. This was based on the theoretical resolution of the array being 0.7Kb, in addition to information from the HapMap phase 1 study showing that approximately 70% of common haplotype blocks are ≤100Kb [8] . Allelic homozygosity was called by the analysis software where there was a stretch of homozygous SNPs in a chromosomal segment.

The second filtering was based on the comparison of all aberrations detected in the patients of this study (N = 35), an in-house patient reference set (N = 35) and an in-house normal reference set (N = 19) as well as the database of genomic variants (DGV) [9]. Aberrations of one patient that were not present in any of the other groups (potentially “unique”) were further processed by studying their genetic content and association to diseases and traits, as stated in publications or OMIM, and whether these correlated to the patient’s phenotype. If this did not yield a candidate aberration, all aberrations of a patient were reviewed based on only the associated OMIM disease, despite the frequency of similar changes in the reference sets. The CNVs that were picked out as potential candidates were further compared to CNV data from 2,065 healthy Finnish individuals. Only those aberrations that were present in less than 50 individuals of the Finnish population cohort were initially considered as potentially pathogenic.

### Validation of candidate aberrations

Only aberrations that were considered to potentially associate with patient’s phenotype were attempted validation.

### Microsatellite marker analysis

Potential segmental UPDs were analyzed by microsatellite marker analysis (chr15: D15S204, D15S124; chr6: D6S468, D6S2418; chr11: D11S4140; chr17: D17S578, D17S1832, D17S1828).

The markers were selected based on their location, and on information that they are highly polymorphic in the Caucasian population. Fragments were labelled with a fluorescent HEX label, and separated on an Applied Biosystem 3730XL (Life Technologies, Carlsbad, CA, USA) capillary electrophoresis instrument, according to manufacturer recommendations. Genotypes were called using Applied Biosystems GeneMapper 3.7 software.

## Results

**Figure 1:**
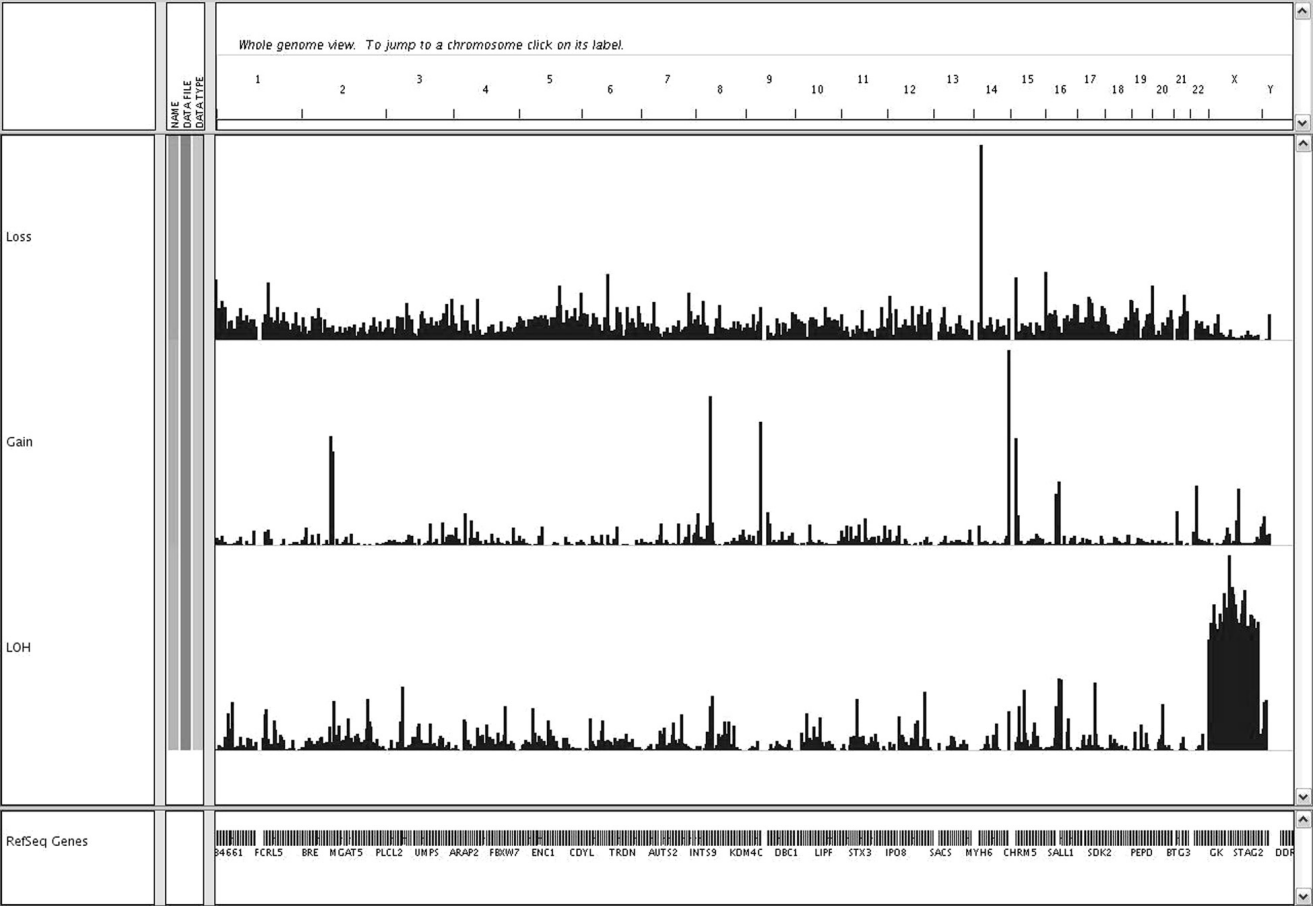
**Frequency of CNVs and allelic homozygosity. **The figure visualizes the frequency of copy number changes (loss and gain) and regions of allelic homozygosity (LOH) in 70 patients (patients of this study N = 35 and the in-house reference set N = 35) with developmental disorders of unknown cause as seen by the Integrative Genomics Viewer (IGV) software V.1.5 (The Broad Institute, Cambridge, MA, USA). The vertical bars show the percentage of patients that have a CNV in a particular area of a chromsome. The higher the bar, the higher the percentage, thus indicating as CNP.

The immense amount of data created by the Genome-wide human SNP array 6.0 warrants filtering for clear interpretation. The Genotyping Console software identified between 200–1000 changes per patient (Figure 1), depending on the technical quality of the result. More changes were detected in samples with lesser quality. After filtering, based on the uniqueness of the CNVs or regions of homozygosity compared to the references, each patient presented 8–20 unique changes (≥90% CNVs) on average. In samples with lesser quality, ≥100 unique changes were detected. Further research on gene content and phenotypes previously mapped to these regions revealed 23 CNVs and 28 regions of homozygosity that putatively correlated with the clinical phenotype in 26 patients. Nine patients had no CNVs or regions of homozygosity spanning known genes or genes known to associate with a disease that correlated with the patient’s phenotype, and their results were thus considered normal. The associated phenotypes related to the aberration found in 26 patients were further evaluated by the patients’ clinicians, and the frequencies of the observed CNVs were monitored in the Finnish population cohort (n = 2,065). As a result, in four patients, a region of allelic homozygosity was considered a potential candidate for causation of their clinical state (Table 2). No CNVs were considered candidates after clinical evaluation.

**Table 2:** Results from microsatellite marker analysis

**Patient**	**Chromosome location**	**Marker**	**Location start**	**Location stop**	**Patient**	**Mother**	**Father**	**Associated OMIMdisease(*gene*)**
1	15q23q24.1	D15S204	72300758	72300879	123/123	123/125	123/125	MIM #209900, Bardet-Biedl Syndrome (*BBS4*)
		D15S124	73092468	73092572	104/106	106/106	104/106	
17	6q16.3	D6S468	101630330	101630479	155/159	159/159	155/155	MIM #611092, Mental retardation (*GRIK2*)
		D6S2418	101352425	101352639	222/230	222/248	230/238	
32	11q13.4	DS11S4140	71945684	71945874	195/195	195/197	195/197	MIM #270400, Smith-Lemli-Opitz syndrome (*DHCR7*)
28	17p13.2p13.1	D17S578	6824007	6824153	173/173	173/173	155/173	MIM #201475, AcylCoA dehydrogenase deficiency (*ACADVL*)
		D17S1832	5972677	5972867	173/185	173/185	171/173/185/193	
		D17S1828	3810467	3810673	220/220	214/220	214/220	

The table presents the results of microsatellite marker analysis of 4 patients and their parents, suggesting biparental inheritance of the genomic segment, and thus exclude segmental UPD. The numbers in the patient/mother/father column represent the two markers detected. In all cases, the patient has likely inherited one marker from each parent.

We further attempted verification of four potential segmental UPDs by microsatellite marker analysis. In all the four cases we observed two distinct alleles with at least some of the more informative multiallelic markers, suggesting that the observed LOHs were most probably caused by the same allelic SNP haplotypes being inherited from both the parents (Table 2).

Further comparison, of all patient SNP array data to the normal data from unaffected individuals, revealed 21 regions of clustering (≥40 % frequency) of allelic homozygosity to specific locations of the genome (Table 3). However, no significant differences were detected between the frequencies of clustered regions in patients and the unaffected relatives in this small set of samples.

**Table 3:** Regions of clustered allelic homozygosity

**Chromosome**	**Band**	**Appoximate range (Kb)**	**Frequency patients (n = 70)**	**Frequency normals (n = 19)**
**1**	p33-p32.3	48 700–53 300	47.7%	47.7%
**1**	q21.1-q21.2	145 800–148 500	49%	50%
**2**	q21.2-q21.3	134 334–136 693	42%	58%
**3**	p21.31-p21.1	46 500–52 500	71%	68%
**4**	p15.1	31 838–34 524	60%	57%
**8**	q22.2	99 200–101 200	47%	63%
**8**	p11.21-p11.1	41 870–43 270	49%	47%
**8**	q11.1-q11.21	47 040–49 000	46%	68%
**10**	p11.21	36 720–38 490	43%	31%
**10**	q22.2-q22.2	73 200–76 460	44%	31%
**12**	q21.32-q21.33	85 850–89 100	47%	47%
**12**	q24.11-q24.13	108 600–111 600	55%	68%
**14**	q23.3-q24.1	65 500–67 100	62%	73%
**15**	q12-q13.1	25 400–27 200	71%	68%
**15**	q15.1-q21.1	40 100–43 730	64%	84%
**15**	q23-q24.1	69 300–71 700	41%	15%
**16**	p11.2-p11.1	33 394–34 550	62%	68%
**16**	q11.2-q12.1	45 092–47 450	64%	63%
**16**	q21-q22.1	64 850–67 100	48%	57%
**17**	q22-q23.2	54 610–56 850	67%	68%
**20**	q11.22-q11.23	31 910–35 500	68%	42%

The table presents the frequency (> 40%) of clustered regions of allelic homozygosity in the patient cohort (n = 70, including in-house reference) compared to the unaffected relatives (n = 19). Kilobase range according to Genome build 19 (NCBI 37).

## Discussion

In our previous study of 150 patients with developmental disorders of unknown cause and a normal karyotype, we were able to identify a (potential) causative aberration in 18% of the patients, by using a 44 K or 244 K array CGH platform [2].

To determine whether, by increasing the resolution, any additional copy number changes or regions of UPD could be detected, we studied 35 patients with a normal array CGH result.

Allelic homozygosity is typically caused by linkage and co-segregation of certain blocks of DNA, termed haplotypes [8]. In a small founder population, such as the Finnish population, the founder effect increases the likelihood that the parents will have the same haplotype, and is as such not a segmental UPD [10]. True segmental UPD is typically due to a duplication in one chromosome and a reciprocal deletion in the other; or the fertilization of a disomic and monosomic gamete, somatic crossing over and subsequent trisomic rescue [11]. If the genomic segment harbours a recessive mutation, which subsequent to UPD will be present in two copies (reduction to homozygosity), it causes a recessive disease. Equally relevant, the segment can be preferentially imprinted, causing complete silencing, which is the equivalent of a deletion. Such presentations of recessive syndromes that are inherited from one normal parent are known in some 40 patients [12].

We were interested to see whether the regions of allelic homozygosity detected by the SNP array were in fact segmental UPDs and associated with an autosomal recessive disease. We were, however, unable to confirm these results and thus the SNP array did not yield more molecular diagnoses in this study of developmental disorders of unknown cause. This may be due to the fact that all patients had previously been studied by another high-resolution array, with 8.9Kb (244 K), 13Kb (180 K), and 35Kb (44 K) theoretical resolutions. Although several new CNVs, previously undetected by the array CGH platform, as well as regions of homozygosity were detected, the pathogenic relevance of these changes were considered insignificant in correlation to the patient’s phenotype. It is, however, possible that changes dismissed in this study are pathogenic by means of spanning genomic segments that do not directly involve disease genes, but rather their regulatory elements.

Our results differ from previously published studies using similar research settings. Bernardini et al. (2010), using the SNP 6.0 array platform with a 75Kb cut-off value for detected CNVs, reported potentially pathogenic CNVs in 6% of patients with normal array CGH result (44 K) [13]. Mannik et al. (2010), using another SNP array with a 50Kb resolution, reported a 23% detection rate in patients with a normal karyotype [14]. Bernardini et al. and Mannik et al. had higher detection rates than this study; perhaps as their first-line of array analysis was, at least partly, done by a lower-resolution method (35Kb and 50Kb respectively) compared to the first-line of detection in this study (8,9Kb, 13Kb and 35Kb). Also, it is important to note that the statistical power is limited by the small size of our patient cohort, and thus results are not entirely comparable with Bernardini and Mannik’s.

UPDs have not been reported in either of the above mentioned studies. However, in a study of 117 patients with a normal karyotype analysed using the 250 K SNP array (Affymetrix, Santa Clara, CA, USA), pathogenic CNVs were detected in 18 patients, and potentially pathogenic segmental UPDs ≥5 Mb in 5, verified by microsatellite marker analysis [15]. The presence of UPDs was also evaluated in another study of 120 patients, using a 500 K SNP array platform [16]. In that study they were unable to verify UPD in any of 121 detected regions of homozygosity in 72 patients with developmental disorder of unknown cause. In addition, in a study of 100 patients with developmental disorder, using a 500 K SNP array, two patients were found to have UPD, the clinical significance of which remained unclear [17]. Thus, UPDs are detectable using SNP arrays, but their clinical significance is difficult to interpret.

## Conclusions

Although there is a clear added value of high-resolution arrays in various fields of genetics, it seems that there is a limit to how much the yield can be increased by increasing the theoretical resolution of the analysis platform. Despite the fact that the SNP array has increased probe spacing compared to the 44 K, 180 K, and 244 K array, and is able to detect more CNVs and regions of homozygosity, interpretation of the vast amount of data and pinpointing of pathogenic changes is difficult. One benefit of using a SNP based platform is the possibility to detect UPDs; however these are relatively rare findings.

This study had a limited number of patients, and so it can only be said that for this study group the optimal yield was conceived when using a resolution of approximately 9Kb [2]. Increasing the resolution beyond that did not confer more diagnoses. It must be emphasized, however, that a larger patient cohort needs to be studied in order to draw final conclusions on the added value of an ultra-high resolution array compared to others. Furthermore, as several reports have shown, some pathogenic aberrations span only a few exons and for detecting such small changes the sensitivity of the SNP 6.0 platform is adequate [18]. It is, however a challenge to filter results correctly and so for diagnostic purposes the choice of platform needs to be carefully considered. Patients with developmental disorders of unknown cause and normal array results may also harbour such small genomic changes (i.e. mutations and unbalanced rearrangements) that are difficult to interpret using microarrays and would require higher resolution methods, such as whole-exome sequencing. Interestingly, a recent study suggests that 80% of patients with a developmental disorder of unknown cause and normal array results can be diagnosed using whole-exome sequencing [19]. Only the future can tell.

### Availability of supporting data

The data set supporting the results of this article is available in the CanGEM repository, http://www.cangem.org/browse.php.

## Competing interests

The authors declare that they have no competing interests.

## Authors’ contributions

LS set up the study, performed the laboratory work and analysis and drafted the manuscript. SA-M helped set up the study and assisted in clinical evaluation of results. TL, KA, MH, LM, and EK, contacted patients and assisted in clinical evaluation of results. IS performed bioinformatics filtering of the results. KK compared CNV findings with data from the Health2000 project. PL performed the microsatellite marker analysis. ML performed analysis for the Health2000 project. JS supervised the performance of the validation studies. SK helped set up and supervise the project. All authors assisted in drafting of the manuscript.

## Pre-publication history

The pre-publication history for this paper can be accessed here:

http://www.biomedcentral.com/1471-2350/13/84/prepub
