# Erratum to: high-resolution SNP array analysis of patients with developmental disorder and normal array CGH result

**DOI:** 10.1186/s12881-014-0124-3

**Published:** 2014-11-18

**Authors:** Linda Siggberg, Sirpa Ala-Mello, Tarja Linnankivi, Kristiina Avela, Ilari Scheinin, Kati Kristiansson, Päivi Lahermo, Marja Hietala, Liisa Metsähonkala, Esa Kuusinen, Maarit Laaksonen, Janna Saarela, Sakari Knuutila

**Affiliations:** Department of Pathology, Haartman Institute, University of Helsinki, and Laboratory of Helsinki and Uusimaa University Hospital, Helsinki, Finland; Rinnekoti Foundation, Rehabilitation Home for Children, Espoo, Finland; Department of Pediatric Neurology, Helsinki University Central Hospital, Helsinki, Finland; Department of Medical Genetics, Väestöliitto, The Family Federation of Finland, Helsinki, Finland; Department of Pathology, VU University Medical Center, Amsterdam, The Netherlands; Public Health Genomics Unit, Department of Chronic Disease Prevention, National Institute for Health and Welfare, Helsinki, Finland; Institute for Molecular Medicine Finland FIMM, University Helsinki, Helsinki, Finland; Department of Clinical Genetics, Turku University Hospital and Department of Medical Biochemistry and Genetics, University of Turku, Turku, Finland; Department of Pediatrics, Satakunta Hospital District, Pori, Finland; Population Health Unit, Department of Health, Functional Capacity and Welfare, National Institute for Health and Welfare, P.O. Box 21, 00014 Helsinki, Finland

## Erratum

After publication of the original article [1] it came to the Publisher’s attention that the several of the article’s authors’ names had been listed incorrectly. The list of authors has now been corrected. We apologise for any inconvenience this has caused.

## References

[1] Siggberg L, Ala-Mello S, Linnankivi T, Avela K, Scheinin I, Kristiansson K, Lahermo P, Hietala M, Metsähonkala L, Kuusinen E, Laaksonen M, Saarela J, Knuutila S (2012). High-resolution SNP array analysis of patients with developmental disorder and normal array CGH results. BMC Med Genet.

